# Polydopamine-tailored paclitaxel-loaded polymeric microspheres with adhered NIR-controllable gold nanoparticles for chemo-phototherapy of pancreatic cancer

**DOI:** 10.1080/10717544.2019.1628118

**Published:** 2019-06-25

**Authors:** Asmita Banstola, Tung Thanh Pham, Jee-Heon Jeong, Simmyung Yook

**Affiliations:** a College of Pharmacy, Keimyung University, Daegu, South Korea;; b College of Pharmacy, Yeungnam University, Gyeongsan, Gyeongbuk, South Korea

**Keywords:** Gold nanoparticle, hyperthermia, pancreatic cancer, photothermal therapy, polydopamine

## Abstract

Chemotherapeutic drugs often used as a first-line treatment of pancreatic cancer (PC) exhibit challenges due to resistance development, lack of selectivity, and tumor heterogeneity. Currently, combination chemo-photothermal therapy is known to enhance the therapeutic efficacy of chemotherapeutic drugs in PC. In this study, we develop adherent gold nanoparticles (GNPs) and paclitaxel (PTX)-loaded PLGA microspheres for the treatment of PC. Polydopamine (pD) was used as a linker to adhere GNPs to the surface of PLGA-Ms and characterized using TEM. Short-term cytotoxicity of GNPs-pD-PTX-PLGA-Ms with or without NIR treatment was evaluated using CCK-8 assays. ROS and western blot assay were performed to determine the intensity of ROS following the treatment of GNPs-pD-PTX-PLGA-Ms with or without NIR in Panc-1 cell line. Successful adhesion of GNPs on the microspheres was confirmed by TEM. CCK-8 assay revealed that GNPs-pD-PTX-PLGA-Ms with NIR showed three-fold higher cytotoxicity, compared to the group without NIR. Furthermore, ROS and western blot assay suggest that GNPs-pD-PTX-PLGA-Ms with NIR showed more ROS generation, followed by downregulation of the expression levels of antioxidant enzyme (SOD2 and CATALASE). These results suggest that the GNPs-pD-PTX-PLGA-Ms in combination with NIR irradiation can provide a synergistic chemo-photothermal therapy for the treatment of PC.

## 1. Introduction

Pancreatic cancer is the fourth most common cause of death in the US, and an estimated 53,600 cases of pancreatic cancer were reported in 2017 in both sexes (Carrera et al., 2017). For most patients diagnosed with pancreatic cancer, curative surgical resection is not an option, and only 20–30% of patients respond to palliative gemcitabine-based chemotherapy (Westphalen et al., 2016). Dense tumor stroma and high interstitial fluid pressure act as barriers for the efficient delivery of the first-line chemotherapeutic drug, gemcitabine, causing chemoresistance. Development of intrinsic, as well as extrinsic resistances, are known to impair the activity of gemcitabine (de Sousa Cavalcante and Monteiro, 2014). To improve the therapeutic outcome in the pancreatic cancer patient, gemcitabine is used alone, or in combination with capecitabine (Herrmann et al., 2007), or platinum-based compound (Heinemann et al., 2008).

Currently, combination therapy to improve the therapeutic outcomes in pancreatic cancer has attracted considerable attention (Xiong et al., 2004; Von Hoff et al., 2011). In particular, the therapeutic efficacy of chemotherapeutic drugs is known to be improved when photothermal therapy (PTT) is used in combination with a strong near-infrared red (NIR) light for conversion of light into heat (Chen et al., 2017). Among several plasmonic photothermal agents, gold nanoparticles (GNPs) have gained popularity for PTT, since they are capable of scattering and absorbing light in the NIR region, and can convert the incident photons into high photothermal energy to induce cell death (Chen et al., 2013; Tran et al., 2016). Compared to the traditional organic dyes, such as rhodamine 6G (Wang et al., 2014) and indocyanine green (Zheng et al., 2011), GNPs offer several-fold higher photo stability (Banstola et al., 2018), and possess high photothermal conversion efficiency (Huang et al., 2008; Emami et al., 2019; Poudel et al., 2019).

PTT is known to be minimally invasive and highly selective when compared to other therapeutic strategies, such as radiation therapy and chemotherapy (Bao et al., 2016; Chen et al., 2015, 2018; Gao et al., 2019; Yu et al., 2019). PTT enhances cancer cell sensitivity, as well as the uptake of chemotherapeutic drugs, by affecting the permeability of cell membrane when NIR is given concomitantly (Chen et al., 2017). In addition, GNPs under NIR irradiation and chemotherapeutic drug would treat tumor leading to cellular apoptosis through several synergistic pathways, such as ROS generation, nuclear damage, and thermal ablation (Thapa et al., 2016a,b).

Despite the noteworthy advantages of GNPs as plasmonic photothermal agents, their clinical application is still controversial, due to their toxicity profile (Cho et al., 2009). Because of their smaller size, free GNPs have wider tissue distribution pattern, and deeper penetration into the tissue (Alkilany and Murphy, 2010). Effective cellular internalization, followed by the accumulation of GNPs in liver and spleen with slower pace of clearance, results in hepatic and splenic toxicity (Chen et al., 2009; Cho et al., 2009; Lim et al., 2011).

Polylactic-co-glycolic acid (PLGA) is one of the most widely used FDA-approved biodegradable polymers, because of its long clinical experience, appropriate degradation characteristics, and availability for sustained drug delivery (Makadia and Siegel, 2011; Ortega-Oller et al., 2015; Bastola et al., 2017). PLGA microspheres have been used as a drug delivery platform for cancer, in order to achieve desirable doses at the tumor site, followed by the sustained release of the drug by intravitreal injection (Thackaberry et al., 2017) and intratumoral injection (Zhang et al., 2018). PLGA microsphere offers several merits: continuous drug release, reduced chance of dose dumping, minimizing of systemic side effects, limited frequency of administration, and therefore, enhanced patient compliance (Zolnik and Burgess, 2007; Wait et al., 2015).

In this study, in order to enhance the efficacy of GNPs as photothermal agent by minimizing their toxicity, PLGA microspheres have been used as a delivery platform for the synergistic PTT of pancreatic cancer. Since PLGA microspheres have large surface area and efficient drug loading capacity, a GNPs-adhered polymeric microsphere based smart drug delivery system was developed for the treatment of pancreatic cancer. Because of the lack of reactivity of surface PLGA microspheres, the PLGA microspheric system is modified using a dopamine polymerization method (Park et al., 2014; Liu et al., 2017). Polydopamine consists of several catechol, amino, and carboxyl functional groups, and is responsible for chelating various metal ions (Ma and Zhang, 2012; Yan et al., 2013). Additionally, the polymerized product has good compatibility, and acts as a good photothermal agent, because of its high photothermal conversion capacity (Zhang et al., 2015; Zheng et al., 2015). Various physicochemical characterization and cellular studies were performed to evaluate the synergistic chemo-photothermal effects of GNPs-adhered and paclitaxel-loaded PLGA microspheres.

## 2. Materials and methods

All materials and methods section are included in Supplementary information.

## 3. Results and discussion

### 3.1. Preparation and characterization of pD-coated and paclitaxel (PTX)-loaded PLGA Ms

**Figure 1.**
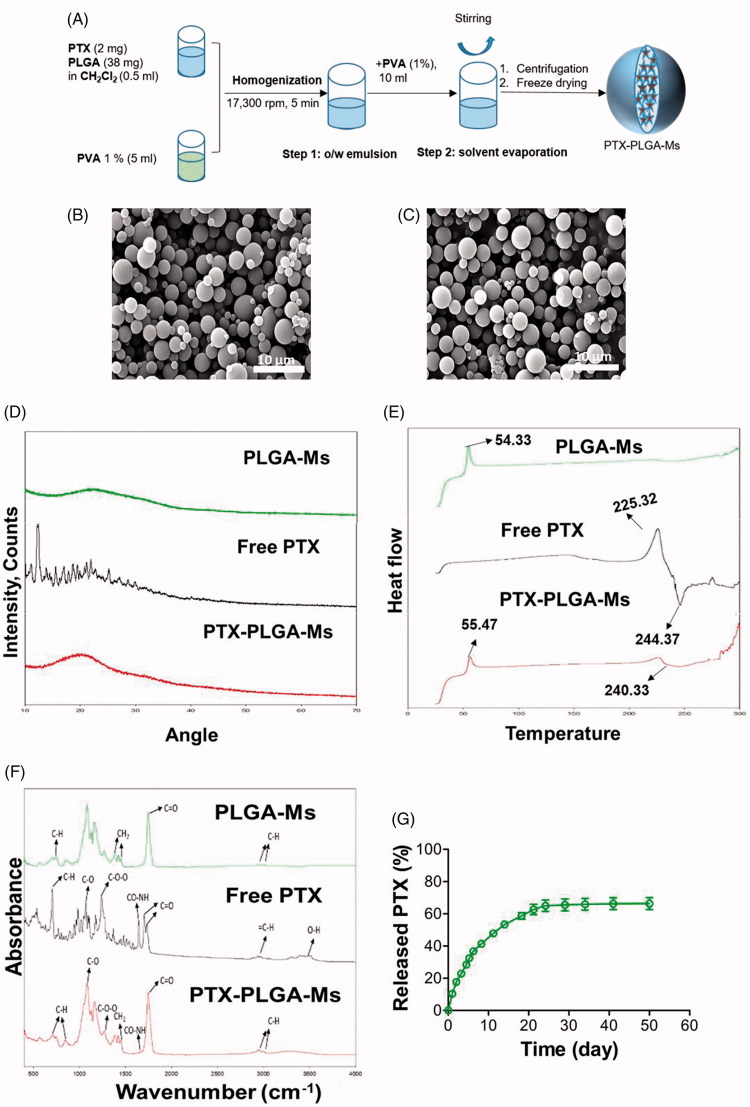
Synthesis and characterization of PTX-PLGA-Ms. (A) Schematic showing the synthesis of PTX-PLGA-Ms. Scanning electron microscopy (SEM) images of (B) PLGA-Ms, and (C) PTX-PLGA-Ms (scale bar: 15 μm). PTX is encapsulated in the polymeric microsphere through hydrophobic interaction. (D) X-ray diffraction (XRD) patterns of PLGA-Ms, free PTX, and PTX-PLGA-Ms. (E) Differential scanning calorimetry (DSC) analysis of the powder form of PLGA-Ms, free PTX, and PTX-PLGA-Ms. (F) Fourier transform infrared spectroscopy (FT–IR) analysis of PLGA-Ms, free PTX, and PTX-PLGA-Ms. (G) Release profile of PTX from PLGA-Ms and pD-PLGA-Ms (*n* = 3). Data are expressed as the mean ± SD.

PTX-loaded PLGA microspheres were prepared using an oil-in-water single emulsion solvent evaporation technique for encapsulating hydrophobic drug, PTX (Figure 1(A)). Figure 1(B) shows the SEM image, which demonstrates the spherical morphology and smooth surface of the microsphere, with the particle size in the range 1–5 µm (Figure 1(B) and Table S1). Figure 1(C) furthermore shows the PTX-loaded PLGA-Ms revealed rough surfaces, due to the rapid evaporation of dichloromethane during the process. Generally, changes in the physical state of the drug have effects on the release pattern and its bioavailability. XRD showed that free PTX has characteristics peaks, most prominently at 2*θ* of 10–30°, suggesting the crystalline nature of the free drug (Figure 1(D)). However, the absence of these characteristic peaks in PTX-PLGA-Ms suggests that PTX was dispersed either in molecular or amorphous state. Furthermore, the decrease in crystallinity of PTX-PLGA-Ms would facilitate the degradation of polymer matrix thereby causing the sustained release of drug over a period of time. In addition, the DSC thermogram of PTX (Figure 1(E)) shows a sharp endothermic peak (244.37 °C) and an exothermic peak (225.32 °C), indicative of its crystalline nature. Moreover, PTX-PLGA-Ms exhibits a broad endothermic peak (240.33 °C), suggesting the amorphous state of drug with improved long-term stability and the bioavailability of drug in the polymeric matrix (Zhang et al., 2018).

Figures 1(F) and S4 show the FT-IR and ^1^H NMR spectra, respectively, which verify the successful encapsulation of PTX in PLGA-Ms. The FTIR spectra of PTX show major infrared peaks at 3415 cm^−1^ (O-H), 2967 cm^−1^ (C–H), 1746 and 1701 cm^−1^ (CO carbonyl ketone group), 1650 cm^−1^ (amide group), 1241 cm^−1^ (C–O–O bending vibrations), 1074 cm^−1^ (C–O stretch vibration), and 702 cm^−1^ (aromatic C–H). The ^1^H NMR signals of PTX observed at (1.44, 1.785, (4.57–4.96) and (7.37–7.94)) ppm reveal the methyl, acetyl, hydroxy, and aromatic protons. PLGA-Ms showed signal arising from methyl (1.46 ppm), methylene (4.91 ppm), and methane (5.2 ppm) protons. Additionally, the FT-IR and ^1^H NMR demonstrate that all the peaks and signals of PTX in PTX-PLGA-Ms were visible, suggesting the successful accommodation of PTX without chemical changes or interaction (Pathak et al., 2017; Zhang et al., 2018).

**Figure 2.**
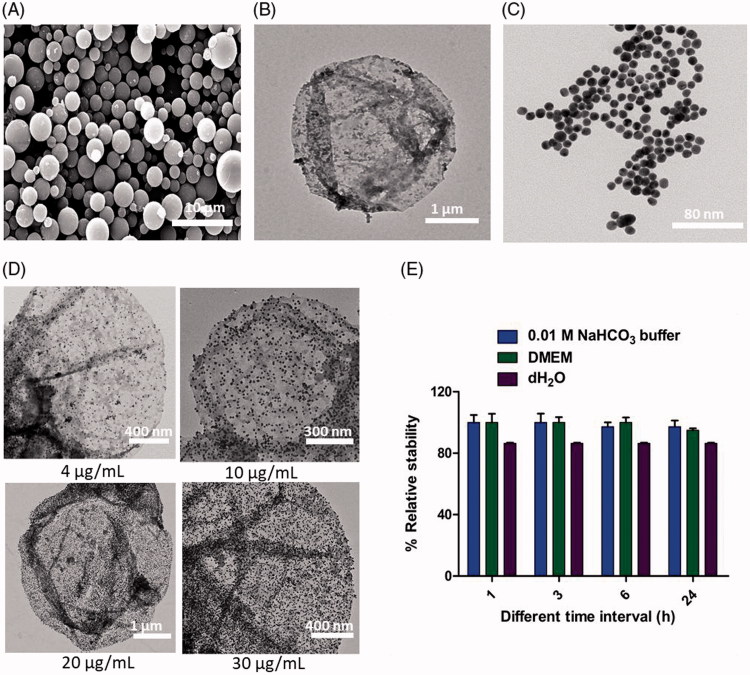
Construction and characterization of GNPs-adhered and PTX-PLGA-Ms (GNPs-PTX-PLGA-Ms). (A) Scanning electron microscopy (SEM) images showing the pD-coated PLGA-Ms. Under weak alkaline condition (0.01 M NaHCO_3,_ pH 8.5), the catechol groups of DOPA can be oxidized to form quinone groups, followed by polymerization giving pD-coated PLGA-Ms. (B) TEM images of pD capsules. (C) TEM image of GNPs (scale bar: 80 nm). GNPs is synthesized using the Turkevich method. (D) TEM images showing the saturation of GNPs adhered to the surface of PTX-loaded PLGA-Ms. The catechol group of polydopamine (pD) chelate GNPs, and form the GNPs-PTX-PLGA-Ms complex. (E) Stability profile of GNPs-pD-PTX-PLGA-Ms after 24 h incubation in 0.01 M NaHCO_3_, DMEM, water.

The surface of PLGA-Ms was modified with dopamine in the presence of alkaline pH 8.5. In an alkaline solution, the oxidized quinone in dopamine reacts with intermolecular catechol/quinone via hydrogen bonding, charge transfer, and π-stacking for dopamine polymerization, thereby forming a thin adherent layer of pD on the surface of PLGA-Ms. This unique property of pD provides the ability for multilayered coating of biomaterials (Hong et al., 2012; Pham et al., 2018). Noteworthy, the SEM image shown in Figure 2(A) reveals that after pD coating, there was no noticeable difference in the surface morphology of pD-PTX-PLGA-Ms, indicating pD coating did not contribute significant mass in comparison to PTX-PLGA-Ms, which was further confirmed by the study provided by Gullotti et al. (2013). In addition, the TEM image shown in Figure 2(B) reveals the successful coating of pD, because of the formation of black layer on the surface of PLGA-Ms.

### 3.2. Synthesis and characterization of GNPs-adhered and PTX-loaded PLGA-Ms

The optimized molar ratio of sodium citrate/HAuCl_4_ (10:1) gives rise to sharp and intense absorption band of GNPs at 520 nm (Figure S1), zeta potential of –29.0 ± 2.2 mV with a mean hydrodynamic diameter of 23.1 ± 3.5 nm (Figure S2). It was noted that the particle size decreased with decreasing molar ratio of sodium citrate which was previously reported from the study of Zabetakis et al. (2012). Figure 2(C) shows the TEM image that reveals spherical and uniform GNPs with an average particle size of 19.5 ± 4.0 nm. Furthermore, Figure S3 exhibits the absence of shift in the surface plasmon resonance peak at 520 nm following post-incubation with 0.01 M NaHCO_3_, pH 8.5 for 24 h suggesting the excellence stability. The UV spectroscopy demonstrated that when 137 µg of GNPs was incubated with 1 mg/mL of PLGA-Ms, 30 µg of GNPs was adhered to the surface of 1 mg/mL of PLGA-Ms (Figure S6). The TEM images showed that 30 µg of GNPs was able to saturate the surface of 1 mg/mL of pD-coated PTX-PLGA-Ms, which was proved from the darker dot present on the surface of the PLGA-Ms, indicating the dense deposition of GNPs on the surface of PTX-PLGA-Ms (Figure 2(D)). This GNPs-Ms interaction is due to the presence of mussel-inspired pD that acts as a linker for the successful adhesion of GNPs to the surface of PTX-PLGA-Ms. The pD consists of catechols group that possess metal-binding ability, resulting in the deposition of adherent and uniform metal coatings onto substrates by electroless metallization (Lee et al., 2007; Gullotti et al., 2013). The GNPs-adhered and PTX-loaded PLGA-Ms was found to be stable, and the stability was above 80% up to 24 h in dH_2_O, DMEM, and 0.01 M NaHCO_3_ respectively (Figure 2(E)). In addition, TEM images of GNPs-adhered and PTX-loaded PLGA-Ms after the incubation in dH_2_O, DMEM, and 0.01 M NaHCO_3_ for 24 h exhibit no obvious deformation and structure damage of the formulations demonstrating the long term stability (Figure S7).

**Figure 3.**
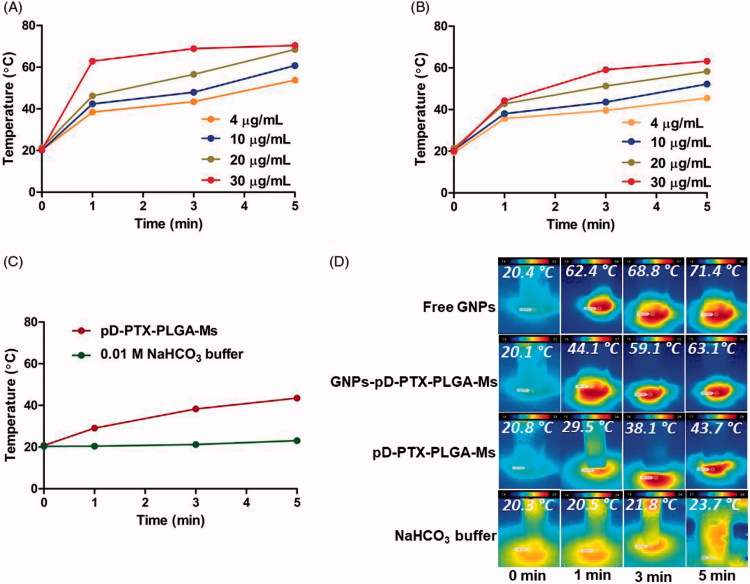
Evaluation of photothermal effect. Photothermal effect measured at (0, 1, 3, and 5) min, after irradiation with the NIR laser at 808 nm with a power density of 2.0 W/cm^2^ (A) Free GNPs, (B) GNPs-pD-PTX-PLGA-Ms, (C) pD-PTX-PLGA-Ms and 0.01 M NaHCO_3_, (D) infrared thermal imaging showing 30 µg/ml of free GNPs, 30 µg/mL of GNP-pD-PLGA-Ms, 1 mg/mL of pD-PLGA-Ms, 0.01 M NaHCO_3_ buffer at (0, 1, 3, and 5) min.

### 3.3. Determination of drug loading, encapsulation efficiency and *in vitro* drug release profile

The HPLC demonstrated that PTX-PLGA-Ms exhibited a loading capacity of 3.5 ± 0.4% and encapsulation efficiency of 70.0 ± 2.6%, as shown in Table S1. The encapsulation efficiency of PTX in PLGA-Ms was high, because of the easier microencapsulation of hydrophobic drug in hydrophobic polymer, due to the reduced likelihood of loss of drug in external aqueous phase via diffusion during the process of microsphere formation, owing to its lipophilicity compared to hydrophilic drug (Pathak et al., 2016). The *in vitro* release profile of PTX from PLGA-Ms showed a release of less than 70% of PTX in 50 d (Figure 1(G)), without any significant initial burst release. This result suggests the controlled release of PTX over a period of time when it is enclosed in the microspheric system. Furthermore, it was also noted that as degradation proceeded (pH 7.4), some surface erosion of PTX-loaded PLGA-Ms was seen at 3 d which was increased with time of incubation (Figure S5). The advantage of this suggested drug delivery platform is to provide controlled and sustained release of drug over a period of time, as well as reduced frequency of dosing (Feng et al., 2014). One of the underlying mechanisms behind the release of PTX from PTX-PLGA-Ms is “drug diffusion,” which indirectly suggests that the PLGA used in this study was suitable for offering a controlled-release carrier matrix for the treatment of pancreatic cancer cells (Chen et al., 2013). Thus, the PTX-PLGA-Ms prepared by our method provide low sustained dose of PTX in the pancreatic tumor, while minimizing systemic exposure, as well as polymeric layers protecting the premature degradation of PTX. Another advantage of this proposed polymeric drug delivery system is that paclitaxel was used in our study as an alternative chemotherapeutic drug to treat pancreatic cancer, but could be replaced with other chemotherapeutic drugs, such as gemcitabine for patients without resistance, which is being used in the clinical setting (Birhanu et al., 2017).

### 3.4. Evaluation of photothermal effect

The photothermal effects of free GNPs, pD-PTX-PLGA-Ms, and GNPs-pD-PTX-PLGA-Ms were evaluated using NIR laser 808 nm (2 W/cm^2^). After the NIR irradiation, the free GNPs showed concentration- and time-dependent rise in temperature (Figure 3(A,D)), which can be attributed to the optical absorption properties of GNPs, thereby converting the NIR light into heat (Rengan et al., 2015). After 5 min irradiation, the Δ*T* of 30, 20, 10, and 4 μg/mL of free GNPs was 71.4, 68.4, 60.7, and 53.8 °C, respectively. The GNPs-pD-PTX-PLGA-Ms (Figure 3(B,D)) also showed the concentration- and time-dependent rise in temperature to a comparable extent of free GNPs reaching up to 63.1 °C after 5 min, indicating that GNPs-pD-PTX-PLGA-Ms were highly photosensitive, and maintained their photothermal effect, even after adhering to the surface of PLGA-Ms for the thermal ablation of the treated pancreatic cancer cells. Moreover, the pD-PTX-PLGA-Ms (Figure 3(C)) also showed a concentration- and time-dependent manner, with Δ*T* of 43.7 °C after 5 min, indicating that pD also has a photosensitive characteristic, and is, therefore, involved in heat generation. However, when the NIR irradiation was carried out on 0.01 M of NaHCO_3_ buffer (Figure 3(C)) only, there was no increase in temperature, demonstrating that the photothermal effects arise only from GNPs and pD.

### 3.5*. In vitro* cell cytotoxicity study

**Figure 4.**
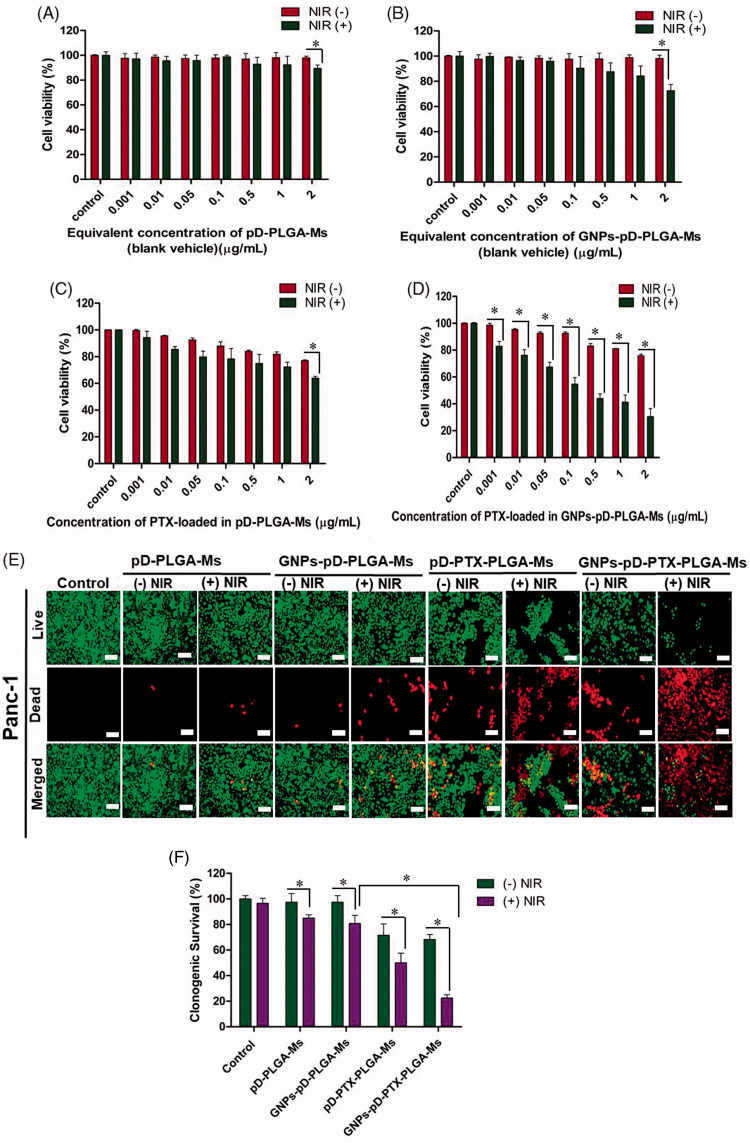
Cell viability studies using CCK-8 assay and irradiation in Panc-1 cells treated with (A) pD-PLGA-Ms, (B) GNPs-pD-PLGA-Ms, (C) PTX-loaded pD-PLGA-Ms, and (D) PTX-loaded GNPs-pD-PLGA-Ms, and (E) live/dead assay using calcein AM (green color) and ethidium homodimer (red color), and (F) clonogenic survival assay after treatment with pD-PLGA-Ms, GNPs-pD-PLGA-Ms, pD-PTX-PLGA-Ms, and GNPs-pD-PTX-PLGA-Ms for 72 h (*n* = 3), with or without NIR irradiation.

Colorimetric assays of different concentrations of pD-PLGA-Ms, GNPs-pD-PLGA-Ms, pD-PTX-PLGA-Ms, and GNPs-pD-PTX-PLGA-Ms were evaluated in Panc-1 cell line. No significant cellular toxicity was observed upon changing the concentration from 0.001 to 2 µg/mL of pD-PLGA-Ms (Figure 4(A)) and GNPs-pD-PLGA-Ms (Figure 4(B)) without NIR irradiation, indicating our blank carrier has no significant unspecific cytotoxicity to the Panc-1 cell. One of the concerns raised in cancer therapy using photothermal agent (a.k.a. GNPs) is non-specific toxicity toward normal cells, liver cells, and spleen cells (Glazer et al., 2011). The free GNPs is a good photothermal agent for cancer treatment, but due to liver and spleen toxicity following intravenous or intraperitoneal administration, its clinical application is as yet very limited (Cho et al., 2009). Chen et al. have demonstrated that because of entrapment in liver and spleen cells, free GNPs is responsible for inducing hepatic and splenic toxicity, followed by disruption of mitochondrial activity (Chen et al., 2009). However, our study has demonstrated that when GNPs are adhered to the surface of microspheres, they have similar capacity to increase local temperature, but we are expecting to have no normal cell, liver, or spleen cell toxicity, because of the colocalization of GNPs-adhered PLGA-Ms on tumor site, thereby reducing the widespread distribution of free GNPs. Thus, GNPs-adhered PLGA-Ms could be an effective photothermal platform for the localized treatment of pancreatic cancer, without inducing toxicity to normal cells. Nevertheless, there was a significant reduction in cell viability after NIR irradiation with pD-PLGA-Ms and GNPs-pD-PLGA-Ms at the concentration of 2 µg/mL (89.50 ± 2.63%; *p* = .007 and 72.55 ± 4.86%; *p* = .001, respectively), in comparison to the group without NIR. In addition, when the NIR is applied, GNPs-pD-PLGA-Ms in comparison to pD-PLGA-Ms caused significant increase in Panc-1 cell cytotoxicity, suggesting the preserved photothermal effect of GNPs even after adhering to the surface of PLGA microsphere, as well as the greater photothermal effect of GNPs than of pD alone. Photothermal therapy utilizes the heat generated by photosensitive agents to induce the photo-ablation of cancer cells. Exposure of GNPs to NIR results in excitation of the surrounding electrons of GNPs. Hot electrons give their energy to the lattice, and through phonon–phonon scattering, heat is transferred to the tumor (Norouzi et al., 2018). This NIR-induced toxicity to pancreatic cancer cells was previously reported by Mocan et al., in which pretreatment with NIR laser-activated GNPs (2 W/cm^2^, 808 nm) enhanced the uptake of GNPs, and induced tumor toxicity in 1.4E7 pancreatic cancer cell line (Mocan et al., 2013). Furthermore, at the concentration of 2 µg/mL, there was significant reduction in cell viability after the incorporation of PTX in pD-PLGA-Ms and GNPs-pD-PLGA-Ms (77.02 ± 0.60%; 1.3-fold, 75.80 ± 1.29%; 1.3-fold, respectively), in comparison to the group without PTX, suggesting the beneficial role of PTX in inhibiting depolymerization of the microtubule spindle, thereby arresting the mitotic cell cycle, ultimately resulting in pancreatic cancer death (Fan, 1999). In addition, compared to the pD-PTX-PLGA-Ms, and GNPs-pD-PTX-PLGA-Ms without NIR, pD-PTX-PLGA-Ms (Figure 4(C)) and GNPs-pD-PTX-PLGA-Ms with NIR treatment (Figure 4(D)) showed a significant dose-dependent cellular cytotoxicity at the concentration of 2 µg/mL (63.92 ± 1.33%; *p* = .0001 and 30.55 ± 5.91%; *p* = .0002, respectively), suggesting the better photothermal effect of the formulation. Furthermore, the addition of the PTX in GNPs-pD–PLGA-Ms with NIR at 2 µg/mL exhibited a dramatic 2.4-fold increase in cellular cytotoxicity, indicating the synergistic chemo-photothermal effect of the final formulation (30.55 ± 5.91%, *p* = .000685). Overall, the treatment with GNPs-pD-PTX-PLGA-Ms and NIR irradiation exhibited the strongest cellular cytotoxicity, with significantly lower viability, compared to photothermal agent or chemotherapeutic agent alone. These results demonstrated the superior effect of the combination therapy on Panc-1 cells, suggesting the chemo-photothermal therapy might sensitize pancreatic cancer cells to the effect of PTX (Zhang et al., 2011). Our study was further supported by Poudel et al. exhibiting that gold nanoshell (GNS) co-delivering two drugs, bortezomib and gemcitabine (GNS-L/GB) together with NIR irradiation showed more pronounced cellular toxicity to pancreatic cell lines (MIA PaCa-2 and Panc-1), than that of drug combination and GNS-L/GB without NIR irradiation (Poudel et al., 2017).

Membrane integrity staining assay was performed to qualitatively determine the cytotoxicity of pD-PLGA-Ms, GNPs-pD-PLGA-Ms, pD-PTX-PLGA-Ms, and GNPs-pD-PTX-PLGA-Ms at the equivalent concentration of 0.1 µg/mL of PTX to Panc-1. The assay demonstrated that Panc-1 cells treated with pD-PLGA-Ms and GNPs-pD-PLGA-Ms without NIR irradiation showed the higher number of live cells, indicated by the strong green fluorescence and bare red color (Figure 4(E)). After NIR irradiation, the number of dead cells was increased, and more pronounced decrease of live cells was observed when GNPs were adhered on the surface of PLGA-Ms, suggesting the stronger photothermal effect of GNPs. Moreover, the addition of PTX in pD-PLGA-Ms and GNPs-pD-PLGA-Ms exhibited an increase in the number of dead cells, and reduction in the number of live cells, compared to the group without PTX. Furthermore, compared to pD-PTX-PLGA-Ms and GNPs-pD-PTX-PLGA-Ms without NIR, pD-PTX-PLGA-Ms and GNPs-pD-PTX-PLGA-Ms with NIR treatment showed a greater number of dead cells and reduction in the number of live cells, revealing the enhanced and improved photothermal effect from GNPs adhered on the surface. In accordance with the results obtained from CCK-8 assay, live/dead assay also demonstrated an increase in the number of dead cells after the treatment with GNPs-pD-PTX-PLGA-Ms with NIR laser, suggesting the superior effect of combinatorial chemo-photothermal therapy, compared with that of single agent (Thapa et al., 2016).

### 3.6. Clonogenic survival (CS) assay

CS assay was carried out to evaluate the long-term cellular cytotoxicity of pD-PLGA-Ms, GNPs-pD-PLGA-Ms, pD-PTX-PLGA-Ms, and GNPs-pD-PTX-PLGA-Ms at the equivalent concentration of 0.1 µg/mL of PTX to Panc-1 cells. This assay revealed that CS of Panc-1 cell exposed to pD-PLGA-Ms and GNPs-pD-PLGA-Ms with NIR irradiation was reduced by 1.1-fold (85.00 ± 2.50%; *p* = .03) and 1.2-fold (80.83 ± 6.29%; *p* = .02), respectively, compared to the group without NIR irradiation (Figure 4(F)). Additionally, a significant reduction in the CS of Panc-1 cell was observed after the incorporation of PTX in pD-PLGA-Ms (1.3-fold, 71.66 ± 8.78%; *p* = .01) and GNPs-pD-PLGA-Ms (1.4-fold, 68.33 ± 3.81%; *p* = .001), compared to the group without PTX. Moreover, after the exposure of pD-PTX-PLGA-Ms and GNPs-pD-PTX-PLGA-Ms with NIR irradiation, the CS of Panc-1 cells was significantly reduced by 1.4-fold (50.00 ± 7.50%; *p* = .03) and 3.0-fold (22.50 ± 2.50%, *p* = .0001), respectively, compared to the group without NIR. Furthermore, there was a dramatic decreased in CS of Panc-1 cell by 5.0-fold (22.50 ± 2.50%, *p* = .0001) after the incorporation of PTX in GNPs-pD-PTX-PLGA-Ms with NIR, indicating the more pronounced long-term cellular cytotoxic effect of synergistic chemo-photothermal therapy. The major underlying reason behind the death of pancreatic cancer patients who have undergone surgery is local recurrence (Manabe et al., 2005). The present study also demonstrates that the combined effects of PTX and GNPs in the presence of NIR decreased the cell density in the colonies, suggesting pronounced long-term cellular cytotoxicity, and also proves that local sustained delivery of PTX from the microspheric system significantly suppressed the tumor growth and regrowth after surgery (Engblom et al., 1996; Kong et al., 2008). This study was further supported by Han et al., in which treatment with hypericin (HY)-functionalized graphene oxide (GO) loaded with doxorubicin (GO-PEG-SS-HY/DOX) and NIR laser (589 nm, 2 W/cm^2^, 5 min) showed the complete absence of the clonogenic formation activity of breast cancer cell lines (MDA-MB-231, MCF-7, and MCF-10A), indicating the synergistic effect of chemo-photothermal therapy reducing the proliferative properties of cancer cells (Han et al., 2018).

### 3.7. Apoptosis assay

**Figure 5.**
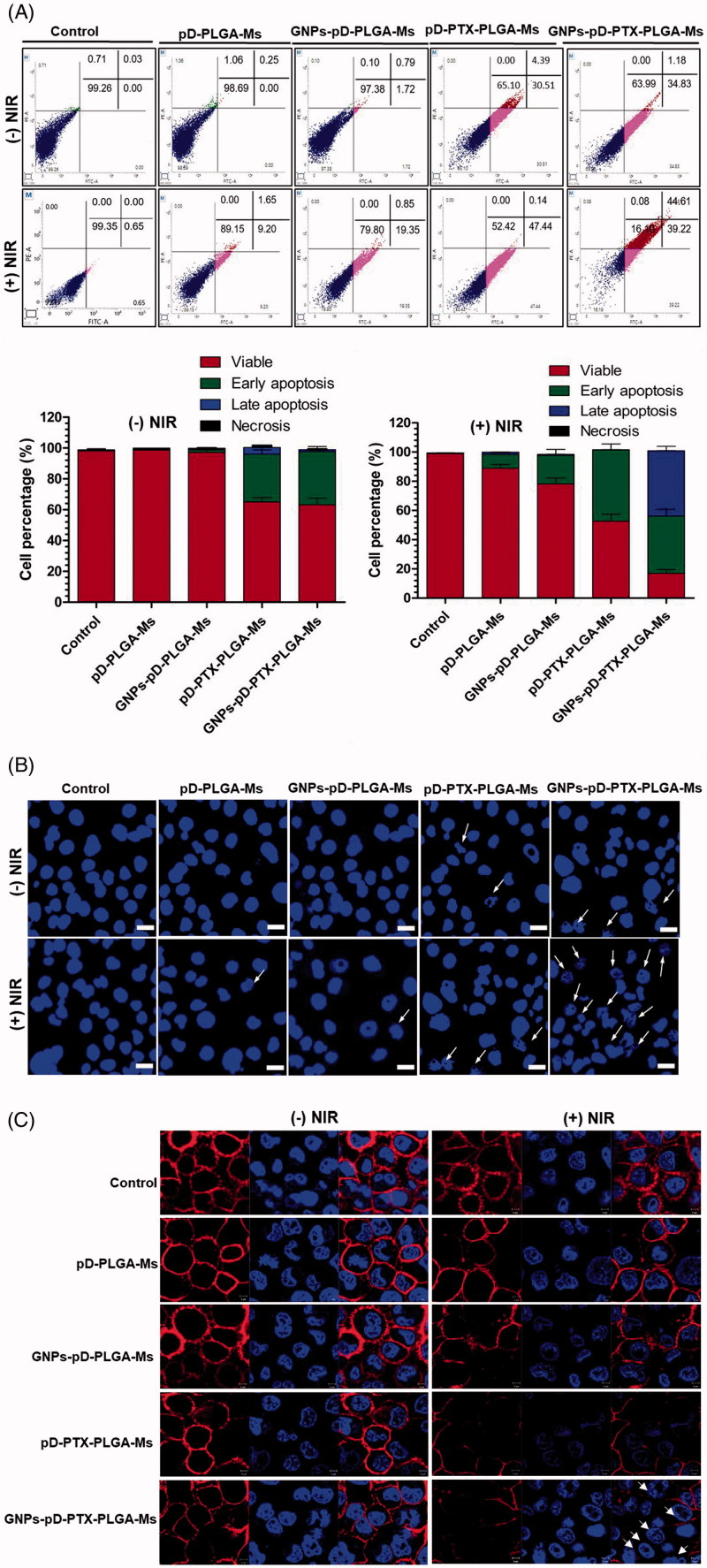
(A) FACS analysis for the determination of the apoptotic effects of GNPs-pD-PTX-PLGA-Ms in Panc-1 cells. NIR treatment consisted of irradiation with an 808 nm NIR laser (2.0 W/cm^2^) for 3 min. (B) Confocal images showing the nuclear apoptosis of GNPs-pD-PTX-PLGA-Ms in Panc-1 cells. NIR treatment consisted of irradiation with an 808 nm NIR laser (2.0 W/cm^2^) for 3 min. (C) Membrane staining assay showing the membrane disruption after incubation of GNPs-pD-PTX-PLGA-Ms in Panc-1 cells with an 808 nm NIR laser (2.0 W/cm^2^) for 3 min.

Flow cytometric analysis of apoptosis was carried out to ascertain the apoptotic mechanism of the underlying synergistic chemo-photothermal effect of GNPs-pD-PTX-PLGA-Ms with or without NIR treatment. Figure 5(A) shows that the lower and upper left quadrants correspond to the percentage of live cells and necrotic cells, whereas the lower and upper right quadrants correspond to the percentages of early and late apoptosis, respectively. Cellular apoptosis assay demonstrated that the apoptosis levels of untreated cells or cells treated with pD-PLGA-Ms (0.00% early apoptosis, and 0.25% late apoptosis) and GNPs-pD-PLGA-Ms (1.72% early apoptosis and 0.79% late apoptosis) were not changed under conditions without NIR exposure (Figure 5(A)). However, the exposures of NIR to the Panc-1 cells treated with pD-PLGA-Ms and GNPs-pD-PLGA-Ms demonstrate cells are undergoing apoptosis (9.20% early apoptosis, and 1.65% late apoptosis; 19.35% early apoptosis, and 0.85% late apoptosis, respectively). Increase in early apoptosis was observed due to the photothermal effect of GNPs and pD, which was further supported by the study of Hao et al., in which the treatment of PLGA@Au nanoparticle after the irradiation with NIR laser (808 nm) increases the percentage of early apoptosis 10.2 ± 2.4% in U87MG glioma cell line (Hao et al., 2015).

The incorporation of PTX in pD-PLGA-Ms and GNPs-pD-PLGA-Ms without NIR enhances the apoptosis proportion (30.51% early apoptosis, and 4.39% late apoptosis; 34.83% early apoptosis, and 1.18% late apoptosis, respectively). More pronounced early apoptosis was observed, due to the incorporation of PTX. This result was further confirmed from the study of Zhang et al., in which free PTX treated against KBv multi-drug resistant tumor cell line caused early apoptosis 6.06 ± 1.13% (Zhang et al., 2011).

Moreover, after the exposure of pD-PTX-PLGA-Ms and GNPs-pD-PTX-PLGA-Ms with NIR irradiation, the apoptosis level of Panc-1 cells was increased (47.44% early apoptosis, and 0.14% late apoptosis; 39.22% early apoptosis, and 44.51% late apoptosis, respectively), in comparison to the group without NIR. More pronounced early apoptosis was observed in both pD-PTX-PLGA-Ms and GNPs-pD-PTX-PLGA-Ms with NIR, indicating heat generated by pD and GNPs under NIR irradiation, as well as chemotherapeutic agent PTX further increasing apoptosis level. This study was further confirmed by the study of Hao et al., in which treatment of PLGA@Au nanoparticle after the irradiation with NIR laser (808 nm) and chemotherapeutic agent DTX further increased the percentage of early apoptosis 15.5 ± 1.6% in U87 MG glioma cell line (Hao et al., 2015). More interestingly, late apoptosis was increased in the GNPs-pD-PTX-PLGA-Ms with the NIR group, suggesting combinatorial chemo-photothermal therapy caused late apoptosis. This result was further supported by Zheng et al., in which treatment with doxorubicin and indocyanine green loaded PLGA–lecithin–polyethylene glycol (PEG) nanoparticles (DINPs) with laser induced more significant late apoptosis (86.9%), as compared with those treated by laser (0.7%) or DINPs (15.3%) (Zheng et al., 2013).

Anticancer chemo-photothermal therapy works by inducing apoptosis in cancer cells, without damaging the surrounding normal cells. Chromatin condensation and the formation of apoptotic body are the identical characteristics of chemotherapeutic agent-induced apoptosis (Liao et al., 2008). These characteristics are observed to be pronounced in cells treated with GNPs-pD-PTX-PLGA-Ms upon NIR irradiation, indicating synergistic anticancer effects, compared with that of chemotherapeutic (PTX) or photothermal agent (GNPs) alone.

Hoechst staining demonstrated that nuclear fragmentation and cellular degeneration result in apoptosis. Figure 5(B) shows that pD-PLGA-Ms and GNPs-pD-PLGA-Ms without NIR did not exhibit nuclear fragmentation, whereas after exposure to NIR, nuclear foci formation was observed. In addition, exposure to pD-PTX-PLGA-Ms and GNPs-pD-PTX-PLGA-Ms with NIR results in a greater proportion of nuclear fragmentation, as well as change in nuclear morphology, in comparison to the group without NIR. However, PLGA-Ms containing PTX and GNPs with NIR showed the maximum apoptotic body formation, followed by a greater formation of nuclear foci than for the group without PTX, suggesting a synergistic chemo-photothermal effect.

### 3.8. Cell membrane staining assay

Without NIR irradiation, no cellular membrane disruption was observed with pD-PLGA-Ms and GNPs-pD-PLGA-Ms, indicating no significant cytotoxic effect of the blank carrier (Figure 5(C)). In contrast, when NIR is given, pD-PLGA-Ms and GNPs-pD-PLGA-Ms showed significant cell membrane disruption. Furthermore, exposure of pD-PTX-PLGA-Ms and GNPs-pD-PTX-PLGA-Ms with NIR exhibited greater damage of the cellular membrane, in comparison to the group without NIR. In addition, the combination of PTX and GNPs together with NIR exhibit more significant disruption of the cell membrane compared to the group without PTX, suggesting the greater cytotoxic effect of combination chemo-photothermal therapy. Localized hyperthermia causes the disruption of cell membrane, followed by the photothermal ablation of cancer cells. In our present study, more pronounced cell membrane disruption was observed when the pancreatic cancer cells were treated with GNPs-pD-PTX-PLGA-Ms upon NIR irradiation, than that of chemotherapeutic agent or photothermal agent alone, suggesting a synergistic effect. These results showed that cell membrane integrity was lost due to the localized hyperthermia of GNPs/light interaction when the GNPs were bound to cell membranes (Sun et al., 2013). The underlying phenomenon behind the loss of membrane integrity is due to certain phenotypic responses, such as the blebbing of membrane, cytoskeletal filaments depolymerization, thermally inactivated membrane proteins and mitochondria, or elevated generation of heat shock proteins (Sun et al., 2013). Our result was further confirmed by Day et al., in which localized hyperthermia (50 mW) was subjected to induce the photothermal ablation of SK-BR-3 breast carcinoma cells, producing extensive membrane blebbing within seconds, leading to cell death (Day et al., 2010).

### 3.9. ROS generation assay

**Figure 6.**
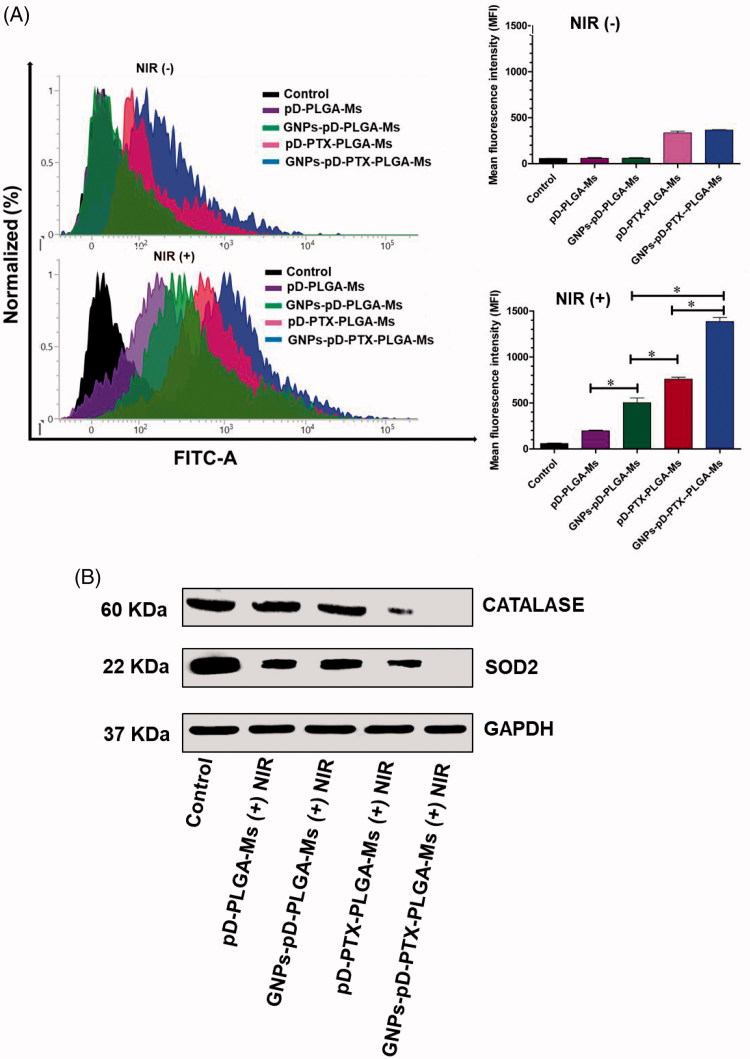
(A) Total ROS production after treatment with GNPs-pD-PTX-PLGA-Ms in Panc-1 cells, followed by the treatment with 808 nm NIR laser (2.0 W/cm^2^) for 3 min and their relative mean fluorescence intensity. (B) Western blot analysis of protein expression in Panc-1 cells, following treatment with 808 nm NIR laser at a power density of 2.0 W/cm^2^ for 3 min.

pD-PLGA-Ms and GNPs-pD-PLGA-Ms with NIR irradiation resulted in 6.7 and 16.7-fold increase in ROS generation, respectively, compared to the group without NIR irradiation, which was mainly associated with the higher ROS generation from GNPs after the irradiation with NIR laser (Figure 6(A)). This result was further supported from the study of Sun et al., in which GNPs irradiated with NIR laser absorbs a quantum of light which then results in excited triplet state thereby entering into two types of a photochemical reaction producing the ROS effect (Sun et al., 2018). Additionally, incorporation of PTX in pD-PLGA-Ms and GNPs-pD-PLGA-Ms without NIR irradiation resulted in 5.7 and 6.1-fold increase in ROS generation, respectively, compared to the group without PTX, suggesting the incorporation of PTX promoted ROS generation through enhancing the activity of NADPH oxidase associated with plasma membranes (Alexandre et al., 2007). In addition, pD-PTX-PLGA-Ms and GNPs-pD-PTX-PLGA-Ms with NIR in comparison to the group without NIR caused (2.3 and 3.8)-fold increase in ROS generation. Furthermore, combining PTX with GNPs after NIR irradiation further enhanced ROS generation (2.7-fold), in comparison to the group without PTX, indicating synergistic chemo-photothermal effect for the induction of cancer cell apoptosis. The appreciable apoptotic activity of GNPs-pD-PTX-PLGA-Ms was mainly associated with the higher ROS generation from GNPs, after the irradiation with NIR laser.

### 3.10. Western blot assay

Compared to control, pD-PLGA-Ms with NIR irradiation downregulates the expression of antioxidant enzymes, SOD2 and CATALASE. Noteworthy, the antioxidant enzyme level was further reduced after the treatment of GNPs-pD-PLGA-Ms with NIR. Furthermore, compared to the photothermal agent alone, the combination of chemo-photothermal agent (pD-PTX-PLGA-Ms and GNPs-pD-PTX-PLGA-Ms) further downregulates the expression of antioxidant enzyme, and thus increases the oxidative stress in Panc-1 cell line (Figure 6(B)). The ROS generation assay results were further supported by the western blot results that present dramatic downregulation in the levels of antioxidant enzyme (SOD2 and CATALASE), upon treatment with GNPs-pD-PTX-PLGA-Ms. These results can be attributed to the combined photothermal effect of GNPs and pD, and the cytotoxic effect of PTX. Generally, for cancer cell survival, there is an upregulation of antioxidant enzyme SOD2 and CATALASE, which are responsible for scavenging ROS, thereby promoting the cellular defense activity (Choi and Kim, 2013). However, our study demonstrated the downregulation of the expression level of antioxidant enzyme after the treatment of different formulations in Panc-1 cell line, which suggests that Panc-1 cell is highly sensitive to the cytotoxic effect of different formulation, and the regulation of SOD2 and CATALASE expression may be an important determinant of the vulnerability of cells to different formulations. This result was further supported by the study of Ding et al., in which the treatment of docosahexaenoic acid (DHA) caused a decrease in the expression of antioxidant enzyme (SOD1) in the human B-cell lymphoma line, which was due to the sensitivity of cell to DHA (Ding et al., 2004).

Recently, treatment of the early stage of pancreatic cancer includes surgery, followed by chemotherapy, radiotherapy, or adjuvant therapy. However, in advanced stage pancreatic cancer, as the tumor cells have disseminated throughout the body, such cases are not suitable for surgery. In addition, the systemic therapies currently been used for pancreatic cancer have largely been disappointing, owing to the poor pharmacokinetic profile, poor penetration of chemotherapeutic agents, as well as tolerance, developed to toxic chemotherapies. If this treatment is successfully translated into the clinic, this can prevent the progression of pancreatic cancer into advanced stage pancreatic cancer, where treatment is extremely difficult. In addition, it can be further applied to the metastatic cancer using the synergy of chemo-photothermal and immune therapy.

## 4. Conclusions

In this study, GNPs-pD-PTX-PLGA-Ms was successfully constructed, and exhibited excellent stability in physiological fluids, making it suitable for *in vivo* delivery. Increased ROS generation, enhanced apoptosis, and NIR-induced photothermal effects, along with the downregulation of antioxidant enzyme, further support the applicability of GNPs-pD-PTX-PLGA-Ms in the treatment of pancreatic cancer. These results suggest that GNPs-pD-PTX-PLGA-Ms could be a promising drug delivery platform for the treatment of locally advanced pancreatic cancer, and should be investigated further, for *in vivo* evaluations. Furthermore, our local delivery platform could be effective in overcoming the limitation associated with the delivery of systemic therapies in PC.

## Supplementary Material

Supplementary_information.docx
