# Supplementary material for: Polydopamine-tailored paclitaxel-loaded polymeric microspheres with adhered NIR-controllable gold nanoparticles for chemo-phototherapy of pancreatic cancer
Source: Drug Deliv. 2019 Jun 25;26(1):629–40. doi: 10.1080/10717544.2019.1628118 (PMC6598510; doi:10.1080/10717544.2019.1628118)
Supplement: Supplementary_information.docx [file IDRD_A_1628118_SM9400.docx]

*Supporting Information*

**Polydopamine Tailored Paclitaxel-Loaded Polymeric Microspheres with Adhered NIR-Controllable Gold Nanoparticles for Chemo-phototherapy of Pancreatic Cancer**

Asmita Banstola^1^, Tung Thanh Pham^2^, Jee-Heon Jeong^2^, Simmyung Yook^1*^

^1^College of Pharmacy, Keimyung University, Daegu 42601, South Korea

^2^College of Pharmacy, Yeungnam University, Gyeongsan, Gyeongbuk 38541, South Korea

**AUTHOR INFORMATION**

Corresponding Author:

* Simmyung Yook, Ph.D., Keimyung University, Daegu 42601, Republic of Korea. Tel: +82–53–580–6656. E–mail: ysimmyung@kmu.ac.kr.

Conflict of interest statement:

The authors declare no competing financial interest

**2. MATERIALS AND METHODS**

**2.1. Materials.** PLGA (PLGA 50:50 DLG 5E) was purchased from Lakeshore Biomaterials Inc., Birmingham, AL, USA). Paclitaxel (PTX) was purchased from TCI Co., Ltd. (Tokyo, Japan). Gold chloride, dopamine hydrochloride, bovine serum albumin, methylene blue, Dichloro-dihydro-fluorescein diacetate (DCFH-DA), 4′,6-diamidino-2-phenylindole (DAPI), dichloromethane, polyvinyl alcohol, sodium bicarbonate buffer, and sodium citrate tribasic dehydrate were obtained from Sigma (St. Louis, MO, USA). Acetonitrile was purchased from Daejung Chemicals and Metals Co., Ltd. (Gyeonggi-do, South Korea). Mounting media was obtained from Vector Laboratories, Inc. (Burlingame, CA, USA). Live/dead assay kit, Hoechst dye, Dil dye, lysis buffer, and Pierce BCA Protein Assay Kit were purchased from Thermo Fisher Scientific (Waltham, MA, USA). Annexin-V-FITC, propidium iodide, and fluorescence-activated cell sorting (FACS) binding buffer were obtained from BD biosciences (San Jose, CA, USA). Fetal bovine serum, sodium pyruvate, and penicillin-streptomycin were purchased from Gibco-Invitrogen (Grand Island, NY, USA). SOD2, Catalase, and GAPDH were purchased from Cell Signaling Technology (Danvers, MA, USA). Column C18 (0.5 μm, (25 cm × 0.46 cm)) was purchased from GL Science (Torrance, CA, USA). Vivaspin (MWCO 300 KDa) was purchased from Sartorius Stedim Lab Ltd. (Stonehouse, UK). Coverslip (24 mm × 24 mm) was obtained from Deckglaser Cover Glasses (Guntersville, Alabama, USA). Cell counting kit (CCK-8) was purchased from Dojindo (Kumamoto, Japan). Tris-buffered saline with 1% Tween 20 (TBST) was purchased from Amresco (Baltimore, USA). Polyvinylfluoride membrane was purchased from Merck Millipore Ltd. (Darmstadt, Germany).

**2.2. Cell lines and culture.** The pancreatic cancer cells, Panc-1, were obtained from the Korean Cell Line bank (Seoul, South Korea). The Panc-1 cells were cultured in Dulbeco’s Modified Eagle’s Media (DMEM) containing 10% of fetal bovine serum and 1% (v/v) penicillin-streptomycin + 2% (v/v) sodium pyruvate. All chemicals were reagent grade, and were used without further purification.

**2.3. Preparation and characterization of polydopamine-coated and paclitaxel-loaded PLGA microsphere.** An emulsion–solvent displacement technique was used to prepare paclitaxel encapsulated PLGA-Ms (PTX-PLGA-Ms) (Achim et al., 2008). Firstly, a mixture of PLGA (38 mg) and PTX (2 mg) was prepared, using dichloromethane (0.5 mL) as a solvent. Afterwards, the resultant solution was added to 1% w/v of polyvinyl alcohol (5 mL). The oil-in-water emulsion was then homogenized at 17,300 rpm for 5 min, followed by magnetic stirring at 2,500 rpm for 4 h, in order to evaporate dichloromethane. Finally, the obtained microspheres were washed 3 times with distilled water, and freeze-dried for long-term storage. Blank microspheres were prepared by the same method, without the addition of PTX. The particle sizes and morphological features of PLGA-Ms and PTX-PLGA-Ms were analyzed by field emission scanning electron microscopy (FE-SEM, H–4100; Hitachi, Tokyo, Japan). Each sample was coated with platinum using an Ion Sputter system, and viewed under FE-SEM operated at acceleration voltage of 10 kV. X-ray diffraction (XRD) patterns of the free PTX and freeze-dried PTX-PLGA-Ms and freeze-dried blank microspheres (PLGA-Ms) were evaluated to determine the physical state of PTX in PTX-PLGA-Ms, using a vertical goniometer and X-ray diffractometry (X’pert PRO MPD diffractometer, Almelo, Netherlands). The diffractometer operated with voltage of 40 kV, current of 30 mA, diffraction angle (2θ) range from 5 to 60°, and scan rate of 5°/min measured Ni-filtered Cu Kα-radiation scattered in the crystalline regions of the sample. Thermal analysis of the powder form of free PTX, PLGA-Ms, and PTX-PLGA-Ms was performed by Differential Scanning Colorimetry (DSC, PerkinElmer DSC 4000, PerkinElmer, Manasquan, New Jersey, USA) to investigate the physical state of drug and long-term stability of drug in the microspheric system. Samples were placed in nitrogen environment, and examined over the temperature range of 0 – 300 ºC with the heating rate of 10 ºC min^-1^*.* Infrared spectroscopy using Fourier transform infrared spectroscopy (FTIR, NICOLET iS10, Thermo Scientific, Waltham, MA, USA) was carried out, in order to examine the encapsulation of drug, as well as chemical changes and chemical interaction of drug in the microspheric system. The powder forms of free PTX, PLGA-Ms, and PTX-PLGA-Ms were taken in KBr disk, and scanning was carried out at the range 400–4,000 cm^-1^. ^1^H nuclear magnetic resonance (NMR) of the free PTX, PLGA-Ms and PTX-PLGA-Ms was measured to determine the drug encapsulation, as well as chemical changes and chemical interaction of drug in the microspheric system. Analysis was performed through JEOL nuclear magnetic resonance (ECZ 500 R, Tokyo, Japan) operated at 500 MHz, using DMSO-D6 as solvent, and tetramethylsilane (TMS) as internal reference. Polydopamine-coated and PTX-loaded PLGA-Ms (pD-PTX-PLGA-Ms) were synthesized by incubating 1 mg/mL of PTX-loaded PLGA-Ms in dopamine hydrochloride (2 mg/mL) dissolved in a 0.01 M sodium bicarbonate buffer (pH 8.5) for 1 h at room temperature with stirring (Zhu et al., 2016). The obtained pD-PTX-PLGA-Ms were centrifuged and lyophilized for GNPs conjugation. The morphological features of pD-coated PTX-loaded PLGA-Ms were analyzed by FE-SEM and transmission electron microscopy (TEM, H7600, Hitachi, Tokyo, Japan).

**2.4. Synthesis and characterization of GNPs-adhered and PTX-loaded PLGA-Ms.** GNPs were prepared by the Turkevich method, with the varying molar ratio of citrate to gold (2:1, 5:1, 10:1, 15:1) (Kimling et al., 2006). Briefly, 10 mg of gold chloride dissolved in 1 mL of distilled water (dH_2_O) was subjected to boiling, and the volume was maintained to 100 mL. Then, different molar ratio of sodium citrate tribasic dihydrate (2:1, 5:1, 10:1, 15:1) was added dropwise to the boiling solution. The mean hydrodynamic diameter of GNPs was measured using dynamic light scattering (DLS, Brookhaven Instruments Corp., version 5.69, USA). The morphological identification of GNPs was carried out using TEM. The localized surface plasmon resonance (LSPR) of GNPs was determined using UV–vis spectroscopy (Shimadzu UV-1800, Kyoto, Japan), scanned at the range between 300 and 700 nm. Stability of the GNPs was also assessed by measuring the time dependent changes in the UV-visible absorption spectra of gold nanoparticles (GNPs) incubated with 0.01 M NaHCO_3_, pH 8.5 for 24 h at room temperature.

GNPs were adhered on the surface of PLGA-Ms using polydopamine (pD) as a linker. Briefly, 1 mg/mL of pD-PTX-PLGA-Ms was incubated with different concentration of GNPs of (34, 67, 103, and 137) µg/mL for 2 h with rigorous vortexing. The obtained GNPs-adhered and PTX-loaded PLGA-Ms (GNPs-pD-PTX-PLGA-Ms) were centrifuged at 2,500 rpm for 5 min. Finally, GNPs-pD-PTX-PLGA-Ms were washed three times with 1 mL of 0.01 M NaHCO_3_. The saturation concentration of GNPs adhered to the surface of PTX-PLGA-Ms was determined by subtracting the amount of GNPs in the supernatant from the initial amount of GNPs used in incubation, with the aid of UV-vis spectrometry. The cross-sectional morphology of GNPs-pD-PTX-PLGA-Ms was examined using TEM. Briefly, the formulation was placed on a carbon-coated copper grid, and then dried under infrared radiation. The grid was then viewed under the accelerating voltage using TEM. The stability of GNPs-pD-PTX-PLGA-Ms was determined in 1 mL of dH_2_O, DMEM, and 0.01 M NaHCO_3_ at 1, 3, 6, and 24 h post-incubation by UV-vis spectrometry. Stability was assessed by subtracting the amount of GNPs adhered to the surface of PLGA-Ms at 0 h from that at 24 h post-incubation. Furthermore, morphological changes of GNPs-pD-PTX-PLGA-Ms was also determined in 1 mL of dH_2_O, DMEM, and 0.01 M NaHCO_3_ after 24 h post-incubation using TEM.

**2.5. Determination of drug loading, encapsulation efficiency, and *in vitro* drug release profile.** Loading capacity (LA) was determined by dissolving 1.8 mg of PTX-PLGA-Ms in 1 mL of acetonitrile to extract drug from the polymeric matrix. The solution was purified by centrifugation at 17,000 rpm for 10 min at 20 °C, and the supernatant was collected for further analysis. The concentration of PTX was determined using the HPLC system (D-2000, Hitachi, Japan) connected to reverse phase analytical column C18. Concentration gradient starting at 50% acetonitrile + 50% dH_2_O, and 90% acetonitrile and 10% dH_2_O at 10 min was used as a mobile phase. A 20 μL sample was injected, and wavelength of 229 nm was used for signal detection. LA and encapsulation efficiency (EE) were calculated using the following formulae:

LA (%) =$\frac{Amount of PTX loaded in Microsphere}{Actual amount of Microsphere}\times100\%$

EE (%) = (1-$\frac{Amount of PTX in supernatant}{Original amount of PTX used})\times100\%$

The *in vitro* release profile of PTX from the polymeric microspheres was assessed in PBS (pH 7.4) under a sink condition. Tween 20 (1%) was added into the PBS (pH 7.4) release medium, to increase the solubility of PTX. An equivalent weight of formulation suspended in release medium was loaded into a vivaspin, and clipped at both ends to ensure no leakage. Afterwards, the vivaspin was kept in 50 mL tube containing 10 mL of release medium. The release study was performed in shaking incubator maintained at 37 °C. At each time point, 1  mL of the release medium was withdrawn, and the release medium was replenished with fresh media. The concentration of PTX was determined by HPLC system, as described in the previous section. The experiment was performed in triplicate. Additionally, in order to investigate the degradation properties of polymeric microsphere after the incubation with PBS (pH 7.4), the samples were dried after each time point and morphological features of PTX-loaded PLGA-Ms were analyzed by FE-SEM.

**2.6. Evaluation of photothermal effect.** In order to evaluate the *in vitro* photothermal effect of the formulation, an 808-nm laser (MDL-H-808 nm-3W-17081128, Changchun New Industries Optoelectronics Tech. Co., Ltd., China) was used. Briefly, 0.01 M NaHCO_3_ buffer, free GNPs, pD-PTX-PLGA-Ms, and GNPs-pD-PTX-PLGA-Ms were suspended in 1 cm cuvettes, and were irradiated with continuous NIR laser (808  nm, 2  W/cm^2^) at different time interval. The temperature in each sample was recorded by thermal camera (Therm-App^®^ TH, Roswell, GA, USA).

**2.7. *In vitro* cell cytotoxicity study.** Panc-1 cells (7,000 cells/200 µL/well) were incubated in a 96-well plate 24 h prior to the experiments, and the different concentrations of pD-PLGA-Ms, GNPs-pD-PLGA-Ms, pD-PTX-PLGA-Ms, and GNPs-pD-PTX-PLGA-Ms were given to the cell. In order to determine the synergistic photo-chemotherapeutic effect, Panc-1 cells were treated with and without near-infrared irradiation (808 nm, 2 W/cm^2^), at a distance of 15 cm for 3 min. After 72 h incubation, cells were washed twice with PBS, and incubated with fresh media overnight. The viability of Panc-1 cells was quantitatively evaluated by a colorimetric assay using CCK-8 assay. Briefly, 10 μL of CCK-8 solution was added to each well, and further incubated for 5 h in the dark. The absorbance from the orange-colored generated formazan was then read at 450 nm using an automated microplate reader (Infinite 200 PRO, TECAN AUSTRIA GmbH, Grodig, Austria). Cell viability was calculated using the following formula:

Cell viability (%) = $\frac{OD450(sample) - OD450(blank)}{OD450(control) - OD450(blank)}$× 100

where, OD is the optical density.

Membrane-integrity staining assay was performed to qualitatively determine the cytotoxicity of pD-PLGA-Ms, GNPs-pD-PLGA-Ms, pD-PTX-PLGA-Ms, and GNPs-pD-PTX-PLGA-Ms at the equivalent concentration of 0.1 µg/mL of PTX to Panc-1, using live/dead assay kit. Briefly, 1×10^5^ Panc-1 cells were incubated in 12-well plate, and the IC_50_ values of pD-PLGA-Ms, GNPs-pD-PLGA-Ms, pD-PTX-PLGA-Ms, and GNPs-pD-PTX-PLGA-Ms were given to the Panc-1 cells with and without NIR irradiation (808 nm, 2 W/cm^2^, 3 min). After 72 h incubation, cells were washed twice with PBS, and stained with calcein AM (2 µM) for staining live cells, and ethidium homodimer (EthD-1) (4 µM) for staining dead cells for 15 min in the dark. Afterwards, cells were washed with PBS, and photographed using a fluorescence microscope (Olympus IX71, Kanagawa, Japan).

**2.8. Clonogenic survival (CS) assay.** The effects of different formulations on the clonogenic survival (CS) of Panc-1 cells were evaluated using CS assay. Panc-1 cells (1×10^5^ cells/mL/well) were plated overnight in a 12-well plate, and cells were treated with pD-PLGA-Ms, GNPs-pD-PLGA-Ms, pD-PTX-PLGA-Ms, and GNPs-pD-PTX-PLGA-Ms for 72 h with/without NIR irradiation (808 nm, 2 W/cm^2^, 3 min), at the equivalent concentration of 0.1 µg/mL of PTX. After washing, cells were harvested, and 100 cells were seeded in a 6-well plate in a triplicate manner, in order to obtain a measurable number of colonies. After 7 days, colonies were stained with methylene blue, and counted manually. The plating efficiency (PE) was calculated from the ratio of number of colonies formed to the number of cells seeded. CS was calculated by dividing the PE of treated cell by the PE of untreated (control cells).

**2.9. Apoptosis assay.** After the incubation of Panc-1 cells (1×10^5^ cells/mL/well) in a 12-well plate overnight, cells were treated pD-PLGA-Ms, GNPs-pD-PLGA-Ms, pD-PTX-PLGA-Ms, and GNPs-pD-PTX-PLGA-Ms with/without NIR irradiation (808 nm, 2 W/cm^2^, 3 min) at the equivalent concentration of 0.1 µg/mL of PTX, to determine the early and late apoptotic effect of our formulations. After 72 h of incubation, cells were washed with PBS, and harvested for further staining with Annexin-V-FITC and propidium iodide for 10 min in the dark. Finally, cells were diluted using FACS binding buffer, and analyzed using FACS Calibur flow cytometer. Nuclear apoptosis study was performed by seeding Panc-1 cells in a 12-well plate containing coverslip for 24 h prior to experiment, and cells were treated with pD-PLGA-Ms, GNPs-pD-PLGA-Ms, pD-PTX-PLGA-Ms, and GNPs-pD-PTX-PLGA-Ms with and without NIR irradiation (808 nm, 2 W/cm^2^, 3 min). After 72 h incubation, cells were washed and fixed with 4% formaldehyde for 10 min. Then, cells were stained with Hoechst dye for 15 min. Afterwards, coverslip containing cells were mounted onto slides containing mounting media, and photographed using confocal laser microscope (CLSM, Leica Microsystem, Wetzlar, Germany).

**2.10. Cell membrane staining assay.** Membrane staining assay was performed, in order to evaluate the disruption of cellular membrane by exposing pD-PLGA-Ms, GNPs-pD-PLGA-Ms, pD-PTX-PLGA-Ms, and GNPs-pD-PTX-PLGA-Ms to Panc-1 cells. Briefly, Panc-1 cells (1×10^5^ cells/mL/well) were seeded in a coverslip placed in 12-well plate. Afterwards, cells were incubated with pD-PLGA-Ms, GNPs-pD-PLGA-Ms, pD-PTX-PLGA-Ms, and GNPs-pD-PTX-PLGA-Ms, followed by NIR irradiation (808 nm, 2 W/cm^2^, 3 min) and further incubated for 72 h. Then the cells were washed with PBS, fixed with 4% formaldehyde solution, and stained with Dil dye for 5 min for membrane staining, and DAPI for 15 min for nuclear staining. Finally, coverslip was mounted onto the glass slide containing mounting media, and observed under CLSM.

**2.11. ROS generation assay.** Hydrogen peroxide level was used to determine the free radical generation by the Panc-1 cells after the treatment with pD-PLGA-Ms, GNPs-pD-PLGA-Ms, pD-PTX-PLGA-Ms, and GNPs-pD-PTX-PLGA-Ms. Initially, 1×10^5^ Panc-1 cells were seeded in a 12-well plate, and allowed to incubate for 24 h. Then, pD-PLGA-Ms, GNPs-pD-PLGA-Ms, pD-PTX-PLGA-Ms, and GNPs-pD-PTX-PLGA-Ms were added to each well with/without NIR irradiation (808 nm, 2 W/cm^2^, 3 min). After incubation for 72 h, cells were washed with PBS, harvested via trypsinization, and stained with freshly prepared DCFH-DA solution for 30 min. Finally, cells were suspended in FACS binding buffer, and analyzed using FACS Calibur flow cytometry.

**2.12. Western blot assay.** Western blot analysis was carried to detect the protein marker, to aid the evaluation of free radical oxygen activity of Panc-1 cells after treatment with pD-PLGA-Ms, GNPs-pD-PLGA-Ms, pD-PTX-PLGA-Ms, and GNPs-pD-PTX-PLGA-Ms. First, Panc-1 cells were seeded in a 6-well plate, and treated with pD-PLGA-Ms, GNPs-pD-PLGA-Ms, GNPs-pD-PTX-PLGA-Ms, and GNPs-pD-PTX-PLGA-Ms with/without NIR irradiation (808 nm, 2 W/cm^2^, 3 min) for 3 days. Afterwards, cells were harvested, lysed with lysis buffer, and the protein concentration was determined using Pierce BCA Protein Assay Kit, according to the manufacturer’s instructions*.* Extracted proteins were separated using 10% Bis-Tris polyacrylamide gel operated at 100 V for 90 min, and then transferred to a polyvinylfluoride membrane. After blocking with 5% bovine serum albumin in TBST, the membrane was incubated overnight with primary antibodies SOD2, Catalase, and GAPDH. Subsequently, the membrane was incubated with secondary antibodies for 1 h, and soaked in chemiluminescent substrate. Protein bands were photographed using Image Quant LAS 4000 (Taunton, MA, USA)

**2.13. Statistical analysis.** Data were expressed as mean ± SD, and statistical significance was tested using a 2-sided Student t-test. The level of significance was set at a P value of < 0.05.


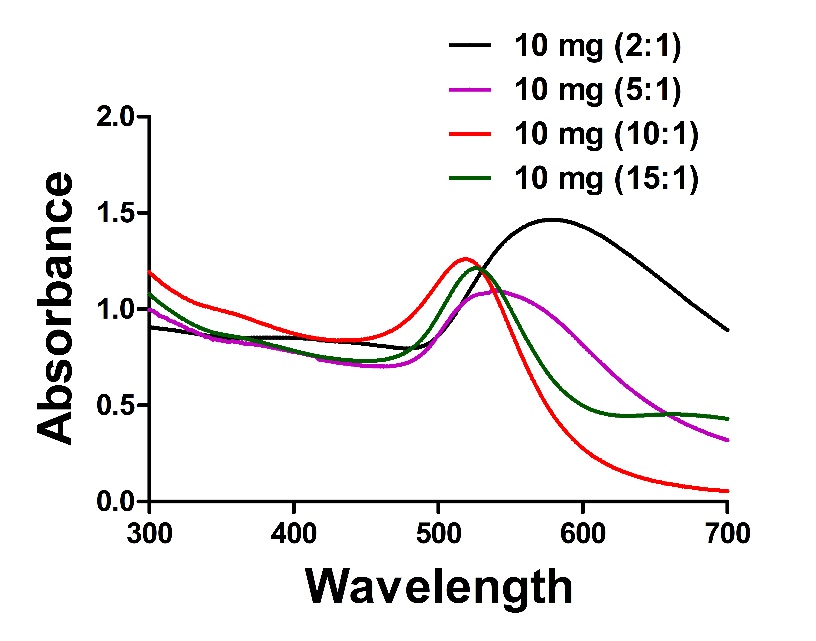


**Figure S1.** UV spectra demonstrating the **o**ptimization of GNPs synthesis from Turkevich method with different molar ratio of sodium citrate/ HAuCl_4._


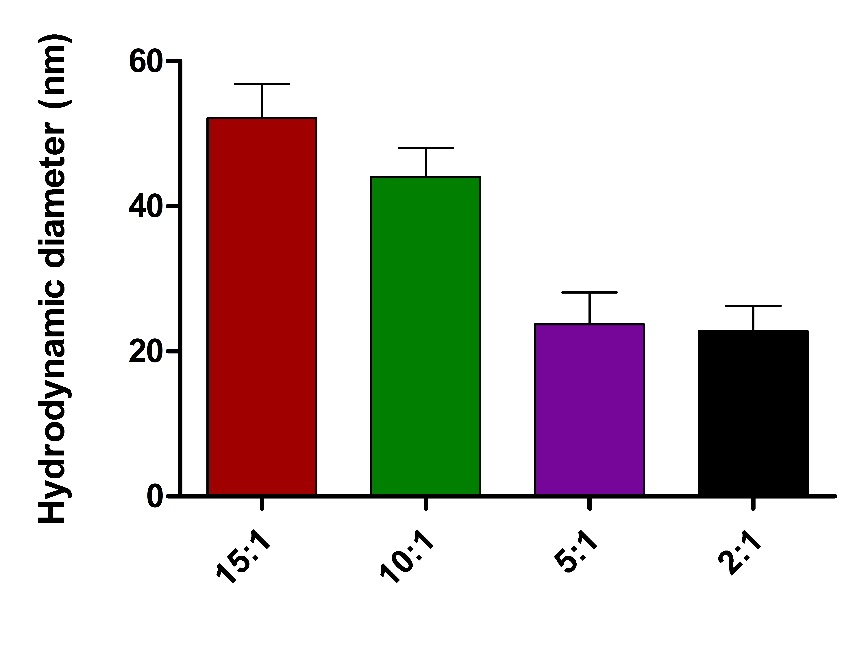


**Figure S2.** Mean hydrodynamic diameter of GNPs with the different molar ratio of sodium citrate/ HAuCl_4._


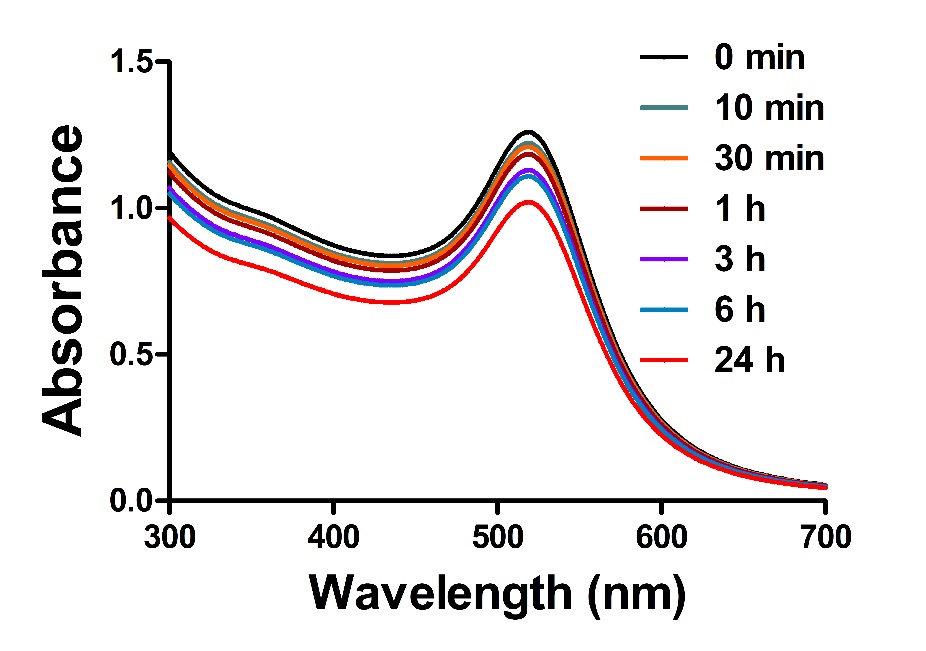


**Figure S3.** Time dependent changes in the UV-visible absorption spectra of gold nanoparticles (GNPs) incubated with 0.01 M NaHCO_3_, pH 8.5 for 24 h at room temperature.


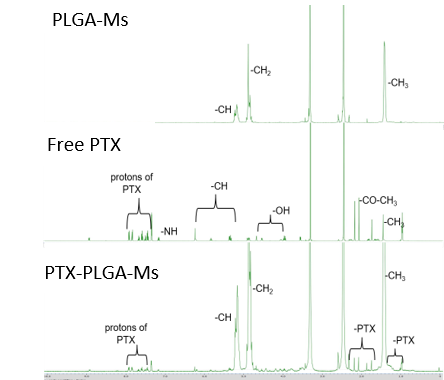


**Figure S4.** ^1^H NMR analysis of PLGA-Ms, free PTX, and PTX-PLGA-Ms.

**(a) (b)**


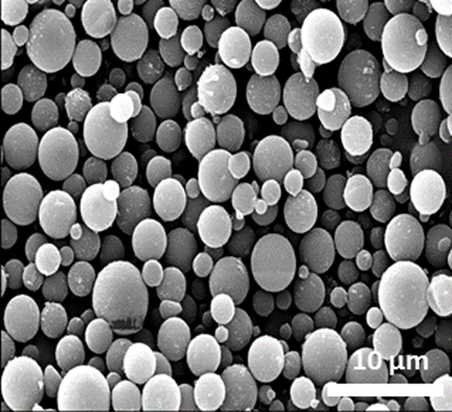

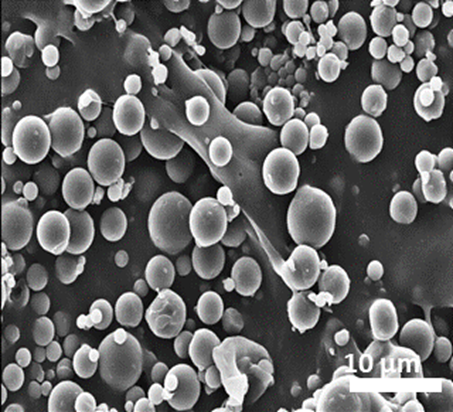

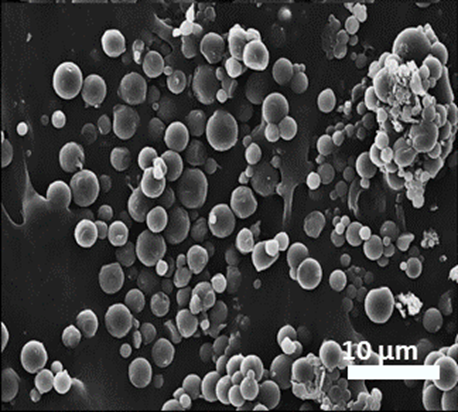

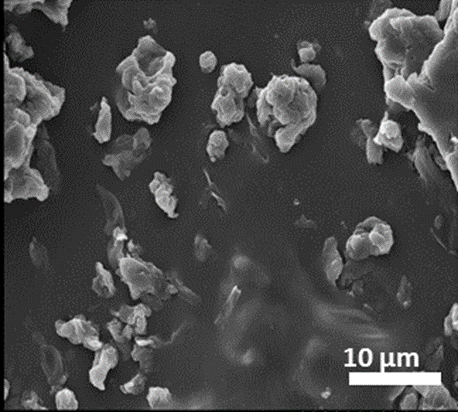


**(c) (d)**

**Figure S5.** SEM image showing the degradation of PTX loaded PLGA-Ms after incubation with PBS, pH 7.4 at **(a)** 0 day, **(b)** 3 day **(c)** 5 day and **(d)** 11 day.


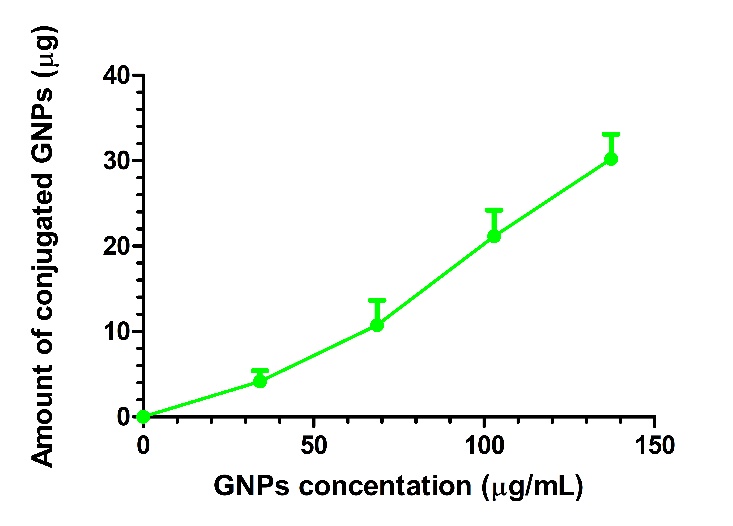


**Figure S6.** Amount of GNPs adhered on the surface of PLGA-Ms determined by UV spectroscopy.

**DMEM**

**Water**

**0.01 M NaHCO_3_**


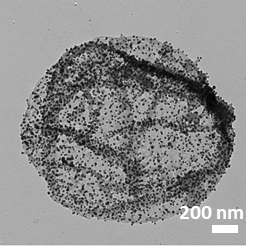

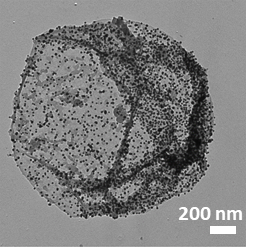

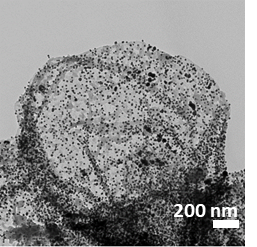


**Figure S7.** TEM image demonstrating the stability profile of GNPs-pD-PTX-PLGA-Ms after 24 h incubation in water, 0.01 M NaHCO_3_ and DMEM respectively.

**Table S1.** Summary of GNPs-pD-PTX-PLGA-Ms explaining the size of PLGA-Ms, loading capacity, and encapsulation efficiency of PTX on PLGA-Ms, and amount of GNPs adhered on PLGA-Ms.

| **Characterization** | **Results** |
| --- | --- |
| Size of PLGA Ms (SEM) | 1-5 µm |
| Loading capacity (L.C) of PTX on PLGA-Ms | 3.5 ± 0.4 % |
| Encapsulation efficiency (E.E) of PTX on PLGA-Ms | 70 ± 2.6% |
| Amount of GNPs required to saturate 1 mg/mL of pD coated PLGA-Ms | 30.0 ± 2.9 µg |

**REFERENCES**

1. Achim M, Tomuta I, Vlase L, Iuga C, Moldovan M, Leucuta SE. (2008). Paclitaxel-loaded poly(lactic-co-glycolic acid) microspheres: preparation and in vitro evaluation. J Drug Deliv Sci Technol 18:410-6.

2. Zhu D, Tao W, Zhang H, et al. (2016). Docetaxel (DTX)-loaded polydopamine-modified TPGS-PLA nanoparticles as a targeted drug delivery system for the treatment of liver cancer. Acta Biomater 30:144-54.

3. Kimling J, Maier M, Okenve B, Kotaidis V, Ballot H, Plech A. (2006). Turkevich method for gold nanoparticle synthesis revisited. J Phys Chem B 110:15700-7.
